# Dental Anxiety in Children With Autism Spectrum Disorder: Understanding Frequency and Associated Variables

**DOI:** 10.3389/fpsyt.2022.838557

**Published:** 2022-04-07

**Authors:** Ye Park, Andrew G. Guzick, Sophie C. Schneider, Madeleine Fuselier, Jeffrey J. Wood, Connor M. Kerns, Philip C. Kendall, Eric A. Storch

**Affiliations:** ^1^Department of Psychiatry and Behavioral Sciences, Baylor College of Medicine, Houston, TX, United States; ^2^Department of Education, University of California, Los Angeles, Los Angeles, CA, United States; ^3^Department of Psychiatry, University of California, Los Angeles, Los Angeles, CA, United States; ^4^Department of Psychology, University of British Columbia, Vancouver, BC, Canada; ^5^Department of Psychology, Temple University, Philadelphia, PA, United States

**Keywords:** autism spectrum disorder, anxiety, dental anxiety, neurodevelopmental disorders, prevalence, ASD

## Abstract

Dental anxiety seems to be elevated in children with autism spectrum disorder (ASD), and may be associated with feelings of helplessness, loss of control, and sensory overload. Dental anxiety, a primary contributor to dental avoidance, can lead to unwanted long-term oral hygiene consequences. This manuscript characterizes the frequency and correlates of dental anxiety in children with ASD. Specifically, this study examined associations between child-reported dental anxiety and parent-reported autism symptom severity, anxiety symptom severity, sensory sensitivity, and internalizing/externalizing symptom severity. Participants included 76 children without cognitive impairment (age in years *M* = 9.9, *SD* = 1.8) who took part in a cognitive behavioral therapy study for children with ASD and co-occurring anxiety disorders. Elevated dental anxiety was found in 68% of participants based on a cut-off score from a dental anxiety measure, with fears related to pain being the most commonly endorsed concern; over half of youth endorsed feeling scared about pinching feelings or having a tooth pulled out at the dentist. No significant correlations between dental anxiety and other variables of interest were found, including overall anxiety severity, ASD symptoms, internalizing and externalizing symptoms, and sensory sensitivities. The findings contextualize the frequency of dental anxiety and its relationship to various variables, which may be useful in tailoring existing treatments to reduce dental anxiety in children with ASD.

## Introduction

Autism spectrum disorder (ASD) is a neurodevelopmental condition that is characterized by social communication deficits as well as repetitive behaviors and restricted interests (1). Mental health disorders occur with high incidence compared to typically developing individuals (2). An estimated 70% of people with ASD experience at least one comorbid mental health disorder, and nearly 40% of individuals may have two or more mental health disorders (3). Among the most common comorbid conditions are anxiety disorders, which occur in over half of children with ASD (4). Anxiety disorders are impairing and impact a number of life domains, including family functioning (5), ASD presentation/severity (6), self-injurious behaviors (5), and social functioning (e.g., peer victimization) (7).

Dental care is one area that may be particularly affected by anxiety among youth with ASD (8), as poor dental hygiene and dental problems are so prevalent in this population. For example, Marshall et al. (9) found that ASD is a strong predictor of having caries, which are permanently damaged areas in teeth that develop into tiny holes. In this study, oral hygiene was the most influential risk indicator associated with the presence of new caries in children with ASD (9). These types of dental issues can lead to pain, poor quality of life, and disease. One of the reasons the ASD population may be more prone to poor dental health is because of dental anxiety.

Children with ASD commonly exhibit uncooperative behaviors during dentist visits, which impedes their oral care and may be tied to anxiety about dental visits and overall oral hygiene (8). Higher rates of dental anxiety in youth with ASD may be due to higher overall anxiety (10), sensory sensitivity (11), and fear of the unfamiliar (12). Individuals with ASD tend to have sensory processing difficulties that lead to over- and under-responsiveness to sensory stimuli and difficulty processing sensory input, a phenomenon associated with significant distress and emotion dysregulation for many youth (13). Sensory sensitivity can involve tactile sensitivity, taste/smell sensitivity, movement sensitivity, visual/auditory sensitivity and more. Stein et al. (8) found that children with ASD exhibited greater behavioral and psychological distress during routine oral care compared to the typically developing group. Behavioral distress was correlated with sensory processing difficulties and sensory sensitivity in the ASD group, suggesting that strategies need to be developed to decrease the distress in children with ASD in dental clinics, given the frequency of sensory sensitivities.

Collaboration between mental health professionals and dentists is crucial to improve the quality and success of oral treatment in children with ASD (14). Dental anxiety is likely a major contributor to difficult behavior at dentist visits and dental avoidance in this population. Though there has been preliminary research in this area, a number of questions remain. In particular, no study has specifically examined youth with co-occurring ASD and anxiety disorders, a population likely to be especially vulnerable to dental anxiety. Furthermore, the degree to which dental anxiety is related to other clinical characteristics among anxious youth remains unclear, as well as the interaction of anxiety and ASD symptom severity.

The aim of this brief report was to characterize the frequency and correlates of dental anxiety in children with ASD and clinically significant anxiety. After describing the frequency of dental fears, the manuscript will examine associations between child-reported dental anxiety and clinical variables expected to be related to dental anxiety across multiple informants (clinicians, parents, and children). It was hypothesized that dental anxiety would be significantly and positively associated with overall anxiety severity, internalizing and externalizing symptoms, sensory sensitivity, and core ASD deficits (10, 11). It was expected that the relationship between overall anxiety and dental anxiety would be particularly pronounced among youth with more severe sensory sensitivity as well as those with more impairing ASD symptoms.

## Methods

### Procedures

The sample consisted of 76 children (age in years *M* = 9.9, *SD* = 1.8) who participated in one site of a previously published three-site randomized controlled trial testing cognitive behavioral therapy (CBT) among 7–13-year-old children with ASD and anxiety and obsessive-compulsive disorders (15). Children included in the study met cut-off criteria for ASD in two measures: the Childhood Autism Rating Scale Second Edition, High Functioning Version [CARS-2HF; (16)] and the Autism Diagnostic Observation Schedule-2 [ADOS-2; (17)]. Participants were excluded from the study if they were receiving concurrent therapy targeting anxiety; had clinically significant suicidality; were diagnosed with lifetime DSM-IV bipolar disorder, schizophrenia, or schizoaffective disorder; or had an initiation or a dosage change of medication weeks before study enrollment. Parents and youth completed a comprehensive assessment before initiating treatment. This study analyzed data from this baseline assessment, including several youth who did not progress in the study due to not meeting inclusion criteria. Please see BLINDED (#) for full inclusion criteria.

### Participants

The majority of the sample was male (75%), consistent with the predominance of ASD in males. The majority of the children identified as White (86%), and most parents were relatively well-educated (48% of fathers and 66% of mothers had at least a 4-year college degree). All participants had an IQ > 70 (*M* = 103.8, *SD* = 14.8) and were able to communicate verbally. See Table 1 for a summary of demographic information.

**TABLE 1:** Demographics.

**Characteristic**	
Female gender, *N* (%)	19 (25%)
Age, M (SD)	9.9 (1.8)
Hispanic/Latinx ethnicity, *N* (%)	11 (15%)
**Race, *N* (%)**	
African American/African	4 (5%)
Asian/Pacific Islander	1 (1%)
White	65 (86%)
Native American or Alaskan	3 (4%)
Other	3 (4%)
**Income total household income, *N* (%)**	
<$40,000	19 (25%)
$40,000–$80,000	20 (27%)
>$80,000	36 (48%)
**Father’s education, *N* (%)**	
≤High school diploma	16 (21%)
Some college	23 (31%)
≥4-year college degree	36 (48%)
**Mother’s education, *N* (%)**	
≤High school diploma	8 (11%)
Some college	18 (24%)
≥4-year college degree	50 (66%)
Autism Diagnostic Observation Schedule-2 total score, M (SD)	14.4 (4.2)
Autism Diagnostic Observation Schedule-2 comparison score, M (SD)	7.9 (2.0)
Pediatric Anxiety Rating Scale Severity, M (SD)	22.3 (3.5)
Estimated full scale intelligence quotient	103.8 (14.8)
Clinically significant dental anxiety, *N* (%)	52 (68%)

### Measures

The following assessments were administered by independent evaluators that were trained by licensed psychologists. Independent evaluators (IE) included advanced graduate students in clinical psychology and postdoctoral fellows. IEs received multiple hours of training and attended weekly supervision sessions with a licensed psychologist experienced with these assessments. Whenever possible, multi-informant assessments of each construct was used.

### Abeer Children Dental Anxiety Scale

The Abeer Children Dental Anxiety Scale (ACDAS) is a 19-item scale of dental anxiety in children that can be completed by children as young as 6. The scale is formed of three components (18). The first two components are completed by the child, where 13 items are related to procedures and sensations during dental visits (The Dental Part) and three items are related to the child’s dental-related cognitions/beliefs (The Cognitive Part). In the third component, the child’s parent/legal guardian establishes whether the child has had previous dental treatment. Additionally, the dentist is asked to rate the child’s behavior when administered in dental settings. For this study, only the Dental Part completed by the child was evaluated. The total scores for The Dental Part of the ACDAS range from 13 to 39, and a cut-off point of ≥26 indicated that the child endorsed dental anxiety (18). This cutoff was determined with receiver operating characteristic analyses among 165 children visiting the dentist, leading to the cut point with optimal sensitivity and specificity in predicting youth with dental anxiety (which was determined with another well-established scale used in that study). The ACDAS demonstrated excellent intra- and inter reliability, good concurrent validity, good discriminant validity and generalizability (18). Internal consistency in this sample was α = 0.80.

### Autism Diagnostic Observation Schedule

The Autism Diagnostic Observation Schedule (ADOS-2) is a widely used and validated semi-structured clinician-rated, observational assessment administered directly to the subject to elicit social interaction and use of language to generate a comparison score that is standardized (17). Five modules are available depending on the individual’s age and language level. Various activities and materials are used to create both structured and unstructured social scenarios. The independent evaluator rates the behaviors and communication that occur during the ADOS-2. The standardized comparison score ranges from 1 to 10, where 10 represents the highest severity of autism-related symptoms (19). The Calibrated Severity Score (CSS) was used for the analysis.

### Child Behavior Checklist

The Child Behavior Checklist (CBCL) is a well-validated 118-item parent-report measure of general emotional and behavioral functioning with excellent psychometric properties, including among youth with ASD (20). For this study, the CBCL for ages 6–18 years was used, and the Internalizing and Externalizing problems scales were used (21).

### Multidimensional Anxiety Scale for Children

The Multidimensional Anxiety Scale for Children (MASC-P) is a parent-report anxiety assessment (22) with excellent psychometric properties in ASD populations (23–25). The MASC is a standardized 39 item measure of anxiety, where each item is rated on a 4-point Likert scale ranging from 0 (never true) to 3 (often true). The four index scores are Social Anxiety, Separation Anxiety, Harm Avoidance, and Physical Symptoms (26), together yielding the Total Score (α = 0.85).

### Pediatric Anxiety Rating Scale

The Pediatric Anxiety Rating Scale (PARS) is a clinician-rated scale assessing anxiety symptoms and the associated severity and impairment in children over the past week. The PARS Severity Scale item scores range from 0 to 5, and a mean of the scores on the seven items was calculated. The PARS has good reliability and validity (27) (internal consistency in this sample: α = 0.66).

### Reynolds Children’s Manifest Anxiety Scale

The Reynolds Children’s Manifest Anxiety Scale (RCMAS-2) is a 37-item self-report instrument designed to assess the level and nature of anxiety. A total anxiety score is calculated based on 28 items (α = 0.93 in this study), which are divided into three anxiety subscales: physiological anxiety (10 items), worry/oversensitivity (11 items), and social concerns/concentration (7 items) (28).

### Short Sensory Profile

The Short Sensory Profile (SSP) is a 38-item parent report form that assesses children’s ability to process sensory information on a 5-point Likert scale. The measures assess children’s response capability to sensory and behavioral/emotional stimuli and their daily performance. Lower scores relate to a greater frequency of sensory symptoms, and higher scores relate to a lower frequency of sensory symptoms (29). The total score represents overall sensory issues across domains (α = 0.87).

### Social Responsiveness Scale

The Social Responsiveness Scale (SRS-P) is a 65-item scale (30) measuring severity of autism spectrum symptoms (e.g., social awareness, autistic preoccupations) for individuals 4–18 years of age with excellent psychometrics (15). The items are scored with a four-point Likert scale ranging from “not true” (score of 1) to “almost always true” (score of 4). The total score showed high internal consistency (α = 0.91).

### Statistical Analysis

Descriptive information on the sample was presented, including anxiety and ASD symptom severity, rates of clinically elevated dental anxiety using an established cutoff (18), and item response distributions on the ACDAS to evaluate the most common areas of dental anxiety. Next, bivariate associations were evaluated between dental anxiety and clinical/demographic variables, including ASD symptom severity, anxiety severity, sensory sensitivity, internalizing symptoms broadly assessed, and total emotional-behavioral problems. With a sample size of 76, assuming significance at α < 0.05 and power = 0.80, this study was powered to detect significant effects of correlation coefficients of at least 0.31. Exploratory analyses evaluated interactions between anxiety severity and sensory sensitivity, as well as anxiety severity and ASD symptoms, to determine whether the impact of anxiety severity on dental anxiety would be moderated by sensory sensitivity or core ASD symptoms. Finally, a multivariate linear regression was planned to be conducted with dental anxiety as the dependent variable, using variables found to have significant bivariate associations as independent variables, in order to identify variables with unique relationships with dental anxiety.

There was one missing MASC and four missing ADOS-2 scores. Patterns of missing data were first analyzed using Little’s test, which found that data were missing completely at random, χ^2^(16) = 11.61, *p* = 0.77, and thus a completer-only approach was used. Data conformed to normal distribution assumptions, with Kolmogorov-Smirnov statistics falling within skewness/kurtosis values of −2 to +2 (31).

## Results

### Frequency of Dental Anxiety Symptoms

Sixty-eight percent (*n* = 52) of participants reported clinically elevated dental anxiety using a previously validated cutoff score from the ACDAS (18). See Table 1 for a summary of demographic information. The most common areas of dental anxiety in this sample were “having a “pinch” feeling in your gum” (58% feeling “scared” about this), “having a tooth taken out” (78%), “having to wear a gas mask” (43%), and “having strange tastes in the mouth” (41%). The most common response to most items assessing how participants would feel at the dentist was “OK.”

### Bivariate Associations Between Dental Anxiety and Other Clinical Variables

Significant associations were not found between the ACDAS and any of the hypothesized variables (see Table 2). Due to this unexpected absence of significant correlations, we further analyzed specific subscales that were hypothesized to be associated with dental anxiety, finding the following correlations: SSP taste/smell (*r* = 0.022, *p* = 0.85), sensation (*r* = −0.20, *p* = 0.087), and SSP-tactile (*r* = −0.039, *p* = 0.74), MASC-harm avoidance (*r* = −0.13, *p* = 0.26), RCMAS-2-worry (*r* = 0.11, *p* = 0.35). Subsequent regressions were not conducted as planned due to the absence of significant bivariate associations.

**TABLE 2:** Item responses to Abeer Child Dental Anxiety Scale (*n* = 76).

	Happy *n* (%)	OK *n* (%)	Scared *n* (%)
(1) Sitting in the waiting room	29 (38%)	33 (43%)	14 (18%)
(2) A dentist wearing a mask on his face	27 (36%)	32 (42%)	17 (22%)
(3) Laying flat on the dental chair	26 (34%)	31 (41%)	19 (25%)
(4) A dentist checking your teeth with a mirror	28 (37%)	34 (45%)	14 (18%)
(5) Having a strange taste in your mouth (e.g., a filling or gloves)	9 (12%)	36 (47%)	31 (41%)
(6) Having a “pinch” feeling in your gum	4 (5%)	28 (37%)	44 (58%)
(7) The feeling up numbness (fat lip/tongue)	8 (11%)	38 (50%)	28 (37%)
(8) A dentist cleaning your teeth by buzzy electric arm that’s spraying water	31 (41%)	27 (36%)	18 (24%)
(9) The sounds that you hear at the dentist	20 (26%)	33 (43%)	22 (29%)
(10) The smell at the dentist	26 (34%)	39 (51%)	11 (15%)
(11) Having a tooth taken out	5 (7%)	12 (16%)	59 (78%)
(12) Wearing a small rubbery mask on your nose to breathe special gas to help you feel comfortable during treatment	12 (16%)	31 (41%)	33 (43%)
(13) Having a “pinch” feeling on the back of your hand	8 (11%)	42 (55%)	26 (34%)

*Items were preceded with the statement, “How do you feel about:”.*

**TABLE 3:** Correlations between clinical variables and ACDAS.

	*r* correlation coefficient
**Anxiety**	
MASC total (parent-report)	0.007
PARS total (clinician-report)	–0.012
RCMAS-2 (child-report)	0.19
**Emotional behavioral symptoms**	
CBCL-internalizing (Parent-report)	0.071
CBCL-total problems (Parent-report)	0.061
**Sensory sensitivity**	
SSP-total score (Parent-report)	–0.10
**ASD symptoms**	
ADOS-comparison (Clinician-report)	0.12
SRS-total score (Parent-report)	0.004
**Interaction terms with anxiety and other factors**	
RCMAS-2 × SRS-total score	0.18
RCMAS-2 × SSP-total score	0.15

*ACDAS, Abeer Child Dental Anxiety Scale; ADOS, Autism Diagnostic Observation Schedule; ASD, Autism spectrum disorder; CBCL, Child Behavior Checklist; MASC, Multidimensional Anxiety Scale for Children; PARS, Pediatric Anxiety Rating Scale; RCMAS-2, Reynolds Children’s Manifest Anxiety Scale; SRS, Social Responsiveness Scale; SSP, Sensory Sensitivity Scale.*

## Discussion

The goal of this study was to examine the frequency of dental anxiety in youth with ASD and anxiety disorders as well as factors related to dental anxiety in this population. Results suggest youth with ASD and co-occurring anxiety may be particularly vulnerable to dental anxiety; the majority of participants (68%) were estimated to experience clinically significant dental anxiety, a pattern that may in turn lead to refusal to attend dental appointments or difficult-to-manage behavior at the dentist, both of which could lead to poor dental health in this population. The most common areas of dental anxiety appear to be related to pain at the dentist, with the majority of participants reporting they are “scared” of pinching feelings in their gums or teeth being pulled out during dental visits. That said, the most common response to most items assessing how children would feel about different aspects of a dental visit was “OK,” suggesting other areas may be less impactful for most youth with ASD and anxiety (e.g., feelings of numbness, noises during dental visits).

Contrary to expectations, there were no significant associations found between dental anxiety and anxiety severity, ASD symptoms, or sensory sensitivities. These findings contrast with prior studies that have found that children with ASD have higher rates of dental anxiety related to their higher overall anxiety severity as well as sensory sensitivities (10), which was not found in this study. There are several reasons this may have been the case. First, it may be that dental anxiety is common among youth with ASD independent of other clinical variables given the novel, uncertain nature of this environment. Fear is a normal reaction for children in novel situations such as dentist visits (32). A limitation of this study is the absence of a neurotypical control group. Comparing youth with ASD with neurotypical youth (with and without clinically significant anxiety) could help determine whether ASD symptoms, anxiety, or both contribute to dental anxiety specifically. The intolerance of uncertainty and novel situations is an important factor to consider when studying dental anxiety in the future. Second, it is possible that no associations were found between dental anxiety and other study variables due to informant effects; there was only one child report measure other than the dental anxiety scale in this analysis (the RCMAS-2) (though it is worth noting no significant association with this variable was found). Regardless, using additional parent- or clinician-report measures of dental anxiety may have provided additional valuable, reliable information. Other well-known and commonly used assessments to measure dental anxiety such as the patient-report Dental Anxiety Scale (33) and the Dental Fear survey (34) could potentially provide more information given that they are longer, more in-depth questionnaires. Although the ACDAS did include parent and dentist sections, those sections of the ACDAS were excluded because those sections are geared toward children who just had a dental visit, which was not the context of this study. Finally, the clinically anxious nature of this sample may have further inhibited an ability to detect significant effects given a potentially limited range of anxiety severity (compared to a sample that recruited youth with and without clinically significant anxiety). Alternatively, it may be that other clinical factors are more highly associated with dental anxiety in this population. In particular, the two items youth most frequently endorsed as “scared” were related to pain (pinching feelings or teeth being pulled). Thus, it may be that other factors (e.g., pain catastrophizing and pain sensitivity) may have been more closely associated with dental anxiety in this sample.

### Clinical Implications and Future Research

Our findings indicate that dental anxiety is common among youth with ASD and anxiety. Understanding dental anxiety in children with ASD more clearly would better equip practitioners to lessen the oral health disparities in children with ASD (35). Future research into dental anxiety in youth with ASD should engage multiple informants, samples with a wider range of anxiety severity, and inclusion of other clinical factors (e.g., pain catastrophizing, distress tolerance, and emotion regulation) should be conducted. Developing this understanding could help inform approaches to tailor existing treatments to reduce dental anxiety in children with ASD.

## Conclusion

The majority of youth with ASD and co-occurring anxiety disorders have significant dental anxiety. Painful dental procedures appear to be the most feared situations among this population. These fears likely present an obstacle to maintaining routine oral care. Future research should further investigate factors associated with dental anxiety and treatment approaches to reduce dental anxiety in children with ASD.

## Data Availability Statement

The original contributions presented in the study are included in the article/supplementary material, further inquiries can be directed to the corresponding author.

## Ethics Statement

The studies involving human participants were reviewed and approved by the University of California, Los Angeles general institutional review board, University of South Florida institutional review board, and the Temple University Human Research Protection Program. Written informed consent to participate in this study was provided by the participants’ legal guardian/next of kin.

## Author Contributions

ES, JW, CK, and PK contributed to the conception and design of the study that this manuscript examined. AG led statistical analyses, contributed to initial drafts, and wrote sections of the manuscript. YP formulated the research question addressed in this manuscript, drafted the first and final versions, and integrated feedback from other authors. All authors contributed to the manuscript revision, read, and approved the submitted version.

## Author Disclaimer

The content is solely the responsibility of the authors and does not necessarily represent the official views of the National Institutes of Health.

## Conflict of Interest

ES received royalties from Elsevier Publications, Springer Publications, American Psychological Association, Oxford, Kingsley, Wiley, Inc., and Lawrence Erlbaum. He holds stock in NView, where he serves on the clinical advisory board. He was a consultant for Levo Therapeutics and is currently a consultant for Biohaven Pharmaceuticals and Brainsway. He co-founded and receives payment from Rethinking Behavioral Health, which is a consulting firm that provides support for implementing evidence-based psychological treatment strategies. PK received royalties from Elsevier Publications, Guilford Publications, Workbook Publishing, and from the translation of his treatment materials. CK receives royalties for an edited book on anxiety and autism published by Academic Press. In addition, she has received honoraria and consulting fees for training other researchers on the Autism Spectrum Addendum.

## Publisher’s Note

All claims expressed in this article are solely those of the authors and do not necessarily represent those of their affiliated organizations, or those of the publisher, the editors and the reviewers. Any product that may be evaluated in this article, or claim that may be made by its manufacturer, is not guaranteed or endorsed by the publisher.
